# Altered Effective Connectivity in the Default Network of the Brains of First-Episode, Drug-Naïve Schizophrenia Patients With Auditory Verbal Hallucinations

**DOI:** 10.3389/fnhum.2018.00456

**Published:** 2018-12-05

**Authors:** Zhiyong Zhao, Xuzhou Li, Guoxun Feng, Zhe Shen, Shangda Li, Yi Xu, Manli Huang, Dongrong Xu

**Affiliations:** ^1^Shanghai Key Laboratory of Magnetic Resonance, East China Normal University, Shanghai, China; ^2^New York State Psychiatric Institute, Columbia University, New York, NY, United States; ^3^Key Laboratory of Brain Functional Genomics (MOE and STCSM), Institute of Cognitive Neuroscience, School of Psychology and Cognitive Science, East China Normal University, Shanghai, China; ^4^College of Medicine, Zhejiang University, Hangzhou, China; ^5^Department of Psychiatry, The First Affiliated Hospital, Zhejiang University School of Medicine, The Key Laboratory of Mental Disorder’s Management of Zhejiang Province, Hangzhou, China

**Keywords:** schizophrenia, auditory verbal hallucinations, resting-state functional magnetic resonance imaging, default mode network, Granger causality analysis, effective connectivity

## Abstract

Although the default mode network (DMN) is known to be abnormal in schizophrenia (SZ) patients with auditory verbal hallucinations (AVHs), it is still unclear whether AVHs that occur in SZ are associated with certain information flow in the DMN. This study collected resting-state functional magnetic resonance imaging data from 28 first-episode, drug-naïve SZ patients with AVHs, 20 SZ patients without AVHs, and 38 healthy controls. We used Granger causality analysis (GCA) to examine effective connectivity (EC) of two hub regions [posterior cingulate cortex (PCC) and anteromedial prefrontal cortex (aMPFC)] within the DMN. We used two-sample *t*-tests to compare the difference in EC between the two patient groups, and used Spearman correlation analysis to characterize the relationship between imaging findings and clinical assessments. The GCA revealed that, compared with the non-AVHs group, EC decreased from aMPFC to left inferior temporal gyrus (ITG) and from PCC to left cerebellum posterior lobe, ITG, and right middle frontal gyrus in SZ patients with AVHs. We also found significant correlations between clinical assessments and mean strengths of connectivity from aMPFC to left ITG and from PCC to left ITG. Moreover, receiver operating characteristic analysis revealed that the above-mentioned effective connectivities had a diagnostic value for distinguishing SZ patients with AVHs from non-AVHs patients. These findings suggest that AVHs in SZ patients may be associated with the aberrant information flows of the DMN, and the left ITG may probably serve as a potential biomarker for the neural mechanisms underlying AVHs in SZ patients.

## Introduction

Auditory verbal hallucinations (AVHs), a symptom that appears to be very pragmatic and is sensitive to the perceptions of sound that occur without corresponding external stimuli, have been demonstrated to exist in 60-90% of schizophrenia (SZ) cases (Alderson-Day et al., 2016). Several studies have examined brain activation in SZ patients with AVHs or hallucination predisposition (Allen et al., 2012; Alderson-Day et al., 2015, 2016). However, the neural basis of this phenomenon is still not well-understood so far.

Resting-state functional magnetic resonance imaging (rs-fMRI) is a task-independent and non-invasive method to assess brain regional and neural circuitry functions (Biswal et al., 1995). According to fMRI findings in the literature, altered resting-state networks (RSNs) have been proposed to involve the psychopathological substrates of SZ (Alderson-Day et al., 2015, 2016; Cui et al., 2017). Default mode network (DMN), one of the most well-known RSNs, seems to play a prominent role in SZ patients (Menon, 2011; Woodward et al., 2011). This large-scale brain network comprises anteromedial prefrontal cortex (aMPFC), posterior cingulate cortex (PCC), and medial temporal lobe including the hippocampus and the lateral temporoparietal area (Buckner et al., 2008). DMN related dysfunction has been reported to be one of the pathomechanisms associated with AVHs in SZ patients (Bastos-Leite et al., 2014; Alderson-Day et al., 2015, 2016). A recent study demonstrated that spatial and temporal instabilities in the DMN were correlated with the severity of hallucinations (Jardri et al., 2013). Another study examined the functional connectivity of two hub regions (PCC and aMPFC) within the DMN and found cross-network abnormalities between DMN and salience system in SZ patients with resistant AVHs compared with those without AVHs (Alonso-Solís et al., 2015). However, findings of changes in the DMN in SZ patients with AVHs were not consistent in the previous reports. Some studies demonstrated increased activations or connectivity in the DMN in SZ patients with hallucinations (Alderson-Day et al., 2015, 2016), whereas other studies found a decrease or no changes in the connectivity (Vercammen et al., 2010; Wolf et al., 2011). Therefore, the role of the DMN in AVHs of SZ patients still remains unclear. Although the variety of results might be related to many factors such as patient characteristics or methodological approaches employed in the analyses.

Effective connectivity (EC) is defined as the influence of one neuronal system on another (Zhao et al., 2016) and is increasingly recognized. Two approaches for studying EC, dynamic causal modeling (DCM) and structural equation modeling (SEM), have been commonly used in SZ patients. For instance, by using DCM, investigators found abnormal EC between anterior cingulate cortex (ACC) and cognition- and emotion-related regions in SZ patients compared with healthy control subjects (HCs) (Cui et al., 2015). In addition, disrupted information flows between thalamus, hippocampus, and auditory cortex were found in SZ patients with AVHs but not in those without AVHs (Li et al., 2017). Using the SEM method, Schlösser et al. (2003) revealed an abnormal pattern of connectivity in the cortical-cerebellar circuit in the SZ patient group relative to the HCs. Moran et al. (2013) identified a diminished positive outflow from dorsal anterior insula to central executive network and DMN in SZ using SEM. However, these models require assumptions about the existence of influence and its directionality between two regions, in which the directionality of the influence defines causality or dependency between the two regions. Therefore, any misspecification of the models may result in erroneous conclusions (Roebroeck et al., 2005; Sathian et al., 2011; Hutcheson et al., 2015). Granger causality analysis (GCA), an EC method that originated from the field of economics, can be used to describe observed data in terms of directed functional interactions or information flow (Zhao et al., 2017). Additionally, the GCA provides information about dynamics and directionality of fMRI BOLD signal in cortical circuits (Liao et al., 2011), which effectively compensates for the shortcomings of the two aforementioned approaches, as no prior knowledge is required (Roebroeck et al., 2005). This method has been employed to detect abnormal EC in SZ patients (Palaniyappan et al., 2013; de la Iglesia-Vaya et al., 2014; Guo et al., 2015). However, the GCA is rarely used to investigate AVHs-related EC changes in first-episode, drug-naïve SZ patients.

In this study, we first used the GCA to detect EC alterations of the DMN in SZ patients with AVHs versus those without AVHs. Later, we performed a Spearman correlation analysis to assess whether the mean strengths of EC correlated with clinical assessments in SZ patients and performed a receiver operating characteristic (ROC) analysis to evaluate the diagnostic value of EC for distinguishing SZ patients with AVHs from non-AVHs patients. This study is an exploratory study. Recent studies of SZ in effective or causal connectivity found that SZ patients exhibited a deceased or increased connectivity in several SZ-related regions or circuits (Guo et al., 2014; Cui et al., 2015; Li et al., 2017), and the findings were not exactly consistent. We therefore hypothesized that compared with the non-AVHs group, the AVHs group would demonstrate altered EC in the DMN, which would be correlated with the clinical symptoms of SZ patients.

## Materials and Methods

### Participants

A total of 48 SZ patients (28 with AVHs and 20 without AVHs) were recruited from the First Affiliated Hospital of Zhejiang University, who fulfilled the diagnostic criteria for SZ mentioned in the Diagnostic and Statistical Manual of Mental Disorders, Fourth Edition (DSM-IV). The Structured Clinical Interview for DSM Disorders (SCID), routine laboratory tests, and physical and neurological examinations were administered for each participant by two clinicians. Inclusion criteria for patients to enter this study were as follows: (1) age between 13 and 45 years; (2) a DSM-IV diagnosis of SZ; (3) first episode of SZ onset; (4) being antipsychotic drug naïve; and (5) of Han nationality. Schizophrenia patients with AVHs required a score of ≥3 on the hallucinatory behavior (P3) item of the Positive and Negative Syndrome Scale (PANSS) (Kay et al., 1987). The recruited patients included both male (*N* = 23; AVH = 13) and female (*N* = 25; AVH = 15) participants. A patient was excluded if any of the following conditions was met: (1) a history or presence of any severe unstable systemic disease; (2) a history of brain tumor, cerebral trauma, seizure disorder, mental retardation, or MRI evidence of structural brain abnormalities; (3) being pregnant, lactating, or planning to be pregnant within the following 6 months; and (4) any contraindication or incompatibility for MRI. Clinical symptoms were quantified with PANSS. Moreover, a sample of 38 age-, gender-, handedness-, and education-matched healthy participants were selected to compose a control group. All participants provided written informed consent in accordance with the Declaration of Helsinki. The Institutional Review Board (IRB) protocol was approved by the Research Ethics Committee of the First Affiliated Hospital, College of Medicine, Zhejiang University.

### MRI Data Acquisition

All data were acquired using a Philips Achieva3.0T TX MRI system (Philips Healthcare, Netherlands), which was equipped with an eight-channel head coil array. The rs-fMRI data were acquired along the axial direction in a sequential mode using a fast field echo-echo planar imaging (FFE-EPI) sequence. The imaging parameters were as follows: 24 slices, repetition time (TR)/echo time (TE) = 2000/35 ms, flip angle (FA) = 80°, slice thickness/gap = 5.0/1.0 mm, voxel size = 2.4 × 2.4 × 5.0 mm^3^, matrix = 100 × 100, and field of view (FOV) = 240 × 240 mm^2^. The rs-fMRI scan lasted for 6 min and 48 s, and we collected a total of 200 image volumes. Additionally, individual three-dimensional high-resolution T1-weighted images were also acquired for the purpose of spatial normalization using a fast field echo sequence: 150 slices, TR/TE = 7.5/3.7 ms, matrix = 240 × 240, slice thickness = 1 mm, FOV = 240 × 240 mm^2^, voxel size = 1 × 1 × 1 mm^3^, and FA = 8°. There were no obvious structural damages on the conventional MRI scans of each participant as examined by two experienced radiologists.

### Data Preprocessing

Preprocessing of the fMRI data was performed using the *Advanced* DPARSF^1^ and SPM8^2^ toolkits. The first 10 functional volumes were discarded to ensure steady state longitudinal magnetization. The remaining 190 volumes were slice-time corrected relative to the middle axial slice to account for the temporal difference in acquisition among the different slices and were then co-registered to correct for head motion during the scan. None of the translation or rotation parameters in any given data set exceeded 2 mm or 2°. Subsequently, white matter, cerebrospinal fluid, and Friston 24-parameter model of head motion (Friston et al., 1996) were removed as nuisance variables. Functional images were spatially normalized to standard stereotaxic coordinates of the Montreal Neurological Institute (MNI) space and resampled into a voxel size of 3 mm × 3 mm × 3 mm. The data were then smoothed by convolution with an isotropic Gaussian kernel at a full width half maximum (FWHM) of 6 mm to decrease spatial noise. Finally, we removed linear trends from the time courses and used temporal band-pass filtering (0.01–0.1 Hz) to remove the effects of low frequency drift and high frequency noise, such as respiratory and heart rhythms.

### Definition of Seed Region of Interest (ROI)

**FIGURE 1:**
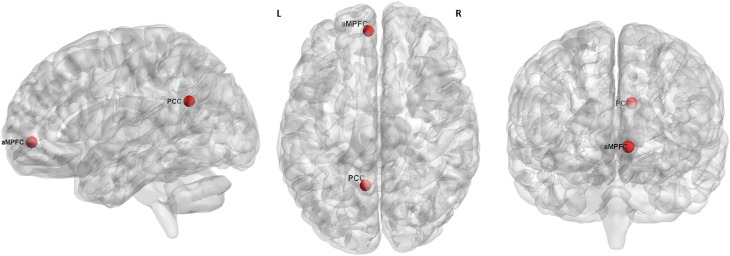
The seed regions (red) for GCA analysis. L, left. R, right. aMPFC, MNI coordinate = –6, 52, –2, radius = 6 mm. PCC, MNI coordinate = –8, –56, 26, radius = 6 mm.

According to the previous reports (Andrews-Hanna et al., 2010; Alonso-Solís et al., 2015), we selected the two hubs of the DMN for resting-state EC analysis, which were defined as a sphere with a radius of 6 mm and centered at the peak MNI coordinates of -6, 52, -2 (aMPFC) and -8, -56, 26 (PCC), respectively (Figure 1).

### Effective Connectivity Analysis

We calculated the EC between the reference time series of the ROI and the time series of each voxel in the whole brain using REST-GCA in the REST toolbox^1^, which is a popular resting-state fMRI data analysis toolkit (Song et al., 2011; Yu et al., 2014; Zuo and Xing, 2014). In addition to EC analysis, this toolkit also includes some other analytic methods, such as functional connectivity analysis based on linear correlation, regional homogeneity, amplitude of low frequency fluctuation (ALFF), and fractional ALFF [For more details related to GCA, see our previous study (Zhao et al., 2016)]. Finally, the GCA maps for all subjects were converted to *z*-values to improve the normality using Fisher’s r-to-z transformation.

### Statistical Analysis

A two-sample *t*-test was used to compare the difference between each pair of all three groups [AlphaSim corrected significance level of *P* < 0.05 (combined voxel height threshold of *P* < 0.005 and an extent threshold of *k* > 69 voxels according a Monte Carlo simulation)] within the gray matter (GM) mask with age, gender, and mean framewise displacement (FD) of Jenkinson (Jenkinson et al., 2002) as covariates. In this study, we primarily focused on the differences in EC in the DMN between AVHs group and non-AVHs group. To examine the correlations between mean EC strength and PANSS scores, a Spearman correlation analysis was performed for each patient group. Additionally, an ROC analysis was performed to assess the diagnostic capability of these imaging measures to distinguish SZ patients with AVHs from those without AVHs. Correlation and ROC analyses were performed using the SPSS software. The threshold of significance was set at *P* < 0.05.

### Validation Analysis

We were also interested in evaluating whether our main results were affected by GM atrophy and by using different preprocessing/analysis strategies (including head motion correction and global signal removal). The relevant procedures are described as follows.

#### The Effects of GM Loss

Previous studies suggested that functional analysis results could potentially be influenced by structural GM differences among groups (Wang et al., 2011; Dai et al., 2014). To explore the possible confounding effect of GM atrophy, we performed a voxel-based morphometry analysis on structural MRI images and took the GM volume (GMV) (i.e., modulated images) as a covariate in the GCA statistical analyses. Briefly, individual GMV maps in the standard space were obtained by a unified segmentation algorithm as described previously (Ashburner and Friston, 2005). After spatially smoothing with a 10-mm FWHM Gaussian kernel, a two-sample *t*-test was performed with age and gender as covariates. The threshold of statistical significance was set at *P* < 0.05 and cluster size > 642 voxels, which corresponded to an Alphasim corrected *P* < 0.05 according to a Monte Carlo simulation within a GM network mask using the dpabi software (Yan et al., 2016). Later, we extracted the averaged GMV in the regions with a significant between-group difference as the covariate to perform the GCA again.

#### The Effects of Head Motion and Global Signal

##### Head motion

Recently, evidence showed that head motion has a confounding effect on functional connectivity analysis (Power et al., 2012; Yan et al., 2013). In this study, we failed to find significant differences in head motion between AVHs group and non-AVHs group [two-tailed two-sample *t*-test: *P* = 0.33 for translational, *P* = 0.11 for rotational, *P* = 0.66 for mean FD of Jenkinson]. Although the Friston 24-parameter model of head motion was regressed in the preprocessing and the statistical analysis was performed by including mean FD as an additional covariate (Yan et al., 2013), to exclude any possible effects of head motion, we re-performed a ‘scrubbing’ procedure on the preprocessed images (Power et al., 2012; Yan et al., 2013). For each subject, rs-fMRI volumes were first censored based on a criterion of FD > 0.2 mm, and the GCA analysis was then reanalyzed using these censored rs-fMRI data.

##### Global signal removal

Currently, it is still controversial whether global signal should be removed in rs-fMRI preprocessing. Several previous studies have suggested that the global signal is associated with non-neuronal activity such as respiration and should be removed (Birn et al., 2006; Chang and Glover, 2009). However, the removal of the global signal introduces widespread negative functional connectivities (Murphy et al., 2009; Weissenbacher et al., 2009). To explore the effects of global signal removal on our results, we reanalyzed our data regressed on the global signal.

## Results

### Characteristics of Participants

No significant differences were detected among the three groups in terms of age, sex, and education level. In addition, the two patient groups had no significant differences in course of illness, age of first onset, and PANSS scores except for PANSS total scores (*p* < 0.05, AVHs > non-AVHs) and PANSS positive scores (*p* < 0.01, AVHs > non-AVHs) (Table 1).

**Table 1:** Demographic and clinical characteristics of patients with AVHs (*n* = 28), patients without AVHs (*n* = 20), and HCs (*n* = 38).

Measure	AVHs	Non-AVHs	HCs
Age (years)^a^	24.32 ± 8.46	24.35 ± 6.94	25.44 ± 7.52
Sex(male/female)^b^	13/15	10/10	17/21
Handedness	R	R	R
Education (years)^a^	11.29 ± 3.00	11.70 ± 2.60	13.34 ± 3.58
Course of illness (months)^a^	17.78 ± 22.57	18.60 ± 15.22	–
Age of first onset (years)^a^	22.75 ± 7.76	22.85 ± 7.69	–
PANSS total scores^a^	88.11 ± 16.89*	78.60 ± 12.55	–
PANSS P scores^a^	23.57 ± 4.66**	18.95 ± 6.37	–
PANSS N scores^a^	20.54 ± 8.58	18.5 ± 7.39	–
PANSS G scores^a^	39.61 ± 7.97	37.10 ± 7.88	–
PANSS S scores^a^	4.39 ± 2.06	4.05 ± 1.73	–

*Data are mean ± SD. ^∗^*p* < 0.05 and ^∗∗^*p* < 0.01 AVHs vs. Non-AVHs. PANSS, Positive and Negative Syndrome Scale. ^*a*^*t*-test. ^*b*^χ^*2*^ test*.

### Effective Connectivity

**FIGURE 2:**
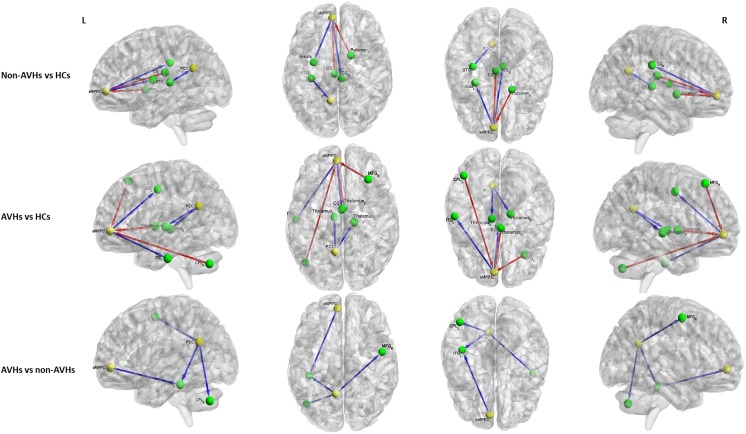
The differences in effective connectivity between any pair of three groups. Yellow ball represents the seed regions and green ball represents the brain regions with significant between-group differences. The blue line represents the decreased EC and the red line represents the increased EC. PCC, posterior cingulate cortex. aMPFC, anteromedial prefrontal cortex. CG, cingulate gyrus. ITG, inferior temporal gyrus. MFG, middle frontal gyrus. CPL, cerebellum posterior lobe. STG, superior temporal gyrus. AVHs, auditory verbal hallucinations. HCs, healthy controls. L, left. R, right.

For the aMPFC, two-sample *t*-tests revealed a significantly lower EC from aMPFC to left inferior temporal gyrus (ITG) in SZ patients with AVHs than those without AVHs. Compared with HCs, the non-AVHs group displayed reduced EC from aMPFC to right cingulate gyrus and left insula and enhanced EC from left cingulate gyrus and right putamen to aMPFC, while SZ patients with AVHs showed a decreased EC from aMPFC to left cingulate gyrus and left ITG and increased EC from right middle frontal gyrus (MFG), right thalamus, and left cerebellum posterior lobe (CPL) to aMPFC (Figure 2 and Table 2).

**Table 2:** Regions showing significant differences in effective connectivity in aMPFC between any pairs of three groups.

Regions	BA	MNI coordinates			Cluster	*t*	AVHs (mean)	Non-AVHs	HCs
		*X*	*Y*	*Z*					
**aMPFC→Other regions**									
***Non-AVHs vs. HCs***									
Right cingulate gyrus	23	6	-27	33	82	-3.63		0.02	0.11
Left insula	13	-30	-6	12	87	-5.52		-0.03	0.01
***AVHs vs. HCs***
Left cingulated gyrus	24	0	-6	45	124	-4.66	0.01		0.07
Left inferior temporal gyrus	20	-57	-18	-33	144	-3.90	-0.01		0.02
***AVHs vs. non-AVHs***
Left inferior temporal gyrus	20	-42	-24	-30	86	-4.18	-0.01	0.03	
**Other regions→aMPFC**									
***Non-AVHs vs. HCs***									
Left cingulate gyrus	23	-3	-21	21	78	4.26		-0.02	-0.07
Right putamen	–	18	3	0	80	4.62		-0.02	-0.09
Right thalamus	–				17				
***AVHs vs. HCs***									
Right middle frontal gyrus	8/9	33	30	54	90	3.76	0.08		-0.04
Right thalamus	–	3	-3	3	81	4.17	-0.02		-0.10
Right putamen	–				10			
Left cerebellum posterior lobe	–	-45	-69	-39	79	3.96	-0.07		-0.09
***AVHs vs. non-AVHs***		
None		

*PCC, posterior cingulate cortex. aMPFC, anteromedial prefrontal cortex. AVHs, auditory verbal hallucinations. HCs, healthy controls*.

For the PCC, relative to those without AVHs, SZ patients with AVHs showed decreased EC from PCC to left CPL, left ITG, and right MFG. Moreover, the non-AVHs group demonstrated reduced EC from left superior temporal gyrus to PCC, and the AVHs group displayed lower EC from PCC to bilateral thalamus when compared with the HCs (Figure 2 and Table 3).

**Table 3:** Regions showing significant differences in effective connectivity in PCC between any pairs of three groups.

Regions	BA	MNI coordinates			Cluster	*t*	AVHs (mean)	Non-AVHs	HCs
		*X*	*Y*	*Z*					
**PCC→Other regions**
***Non-AVHs vs. HCs***									
None									
***AVHs vs. HCs***									
Left thalamus	–	-9	-15	3	101	-4.57	-0.05		0.01
Right thalamus	–				69				
***AVHs vs. non-AVHs***									
Right middle frontal gyrus	6	51	-3	54	114	-3.64	-0.05	0.02	
Left inferior temporal gyrus	20	-42	-33	-21	98	-4.35	-0.02	0.02	
Left cerebellum posterior lobe	–	-39	-57	-57	95	-4.29	-0.02	0.02	
**Other regions→PCC**									
***Non-AVHs vs. HCs***									
Left superior temporal gyrus	41	-33	-27	9	115	-4.54		-0.04	0.03
***AVHs vs. HCs***									
None									
***AVHs vs. non-AVHs***									
None									

*PCC, posterior cingulate cortex. aMPFC, anteromedial prefrontal cortex. AVHs, auditory verbal hallucinations. HCs, healthy controls*.

### Correlation and ROC Analysis

**FIGURE 3:**
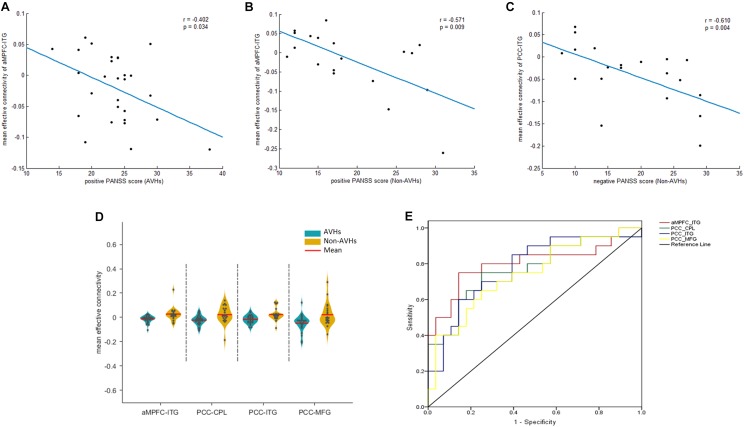
Correlation and ROC analysis results. The significant correlations between mean EC and clinical assessments **(A–C)**. The quantitative display of mean ECs in the four regions with between-groups differences **(D)** and ROC results **(E)**. PCC, posterior cingulate cortex. aMPFC, anteromedial prefrontal cortex. ITG, inferior temporal gyrus. MFG, middle frontal gyrus. CPL, cerebellum posterior lobe. CPL, cerebellum posterior lobe. AVHs, auditory verbal hallucinations. L, left. R, right.

We extracted the average EC in the four regions that displayed a significant difference between the two patients groups. Later, a Spearman correlation analysis was performed between mean EC and duration of illness as well as PANSS scores across all the patients. Significant negative correlations were observed between the EC from aMPFC to left ITG and the PANSS positive scores (*r* = -0.402, *p* = 0.034) (Figure 3A) in the AVHs group. Significant negative correlations, between (1) the EC from aMPFC to left ITG and the PANSS positive scores (*r* = -0.571, *p* = 0.009) (Figure 3B) and (2) the EC from PCC to left ITG and the PANSS negative scores (*r* = -0.610, *p* = 0.004) (Figure 3C), were both observed in the non-AVHs group (uncorrected). However, these correlations failed to survive when the multiple comparisons were corrected for *p* < 0.05 by FDR, perhaps owing to the limited statistical power provided by the limited size of the samples. Additionally, we displayed the mean strength for each EC with between-group difference (Figure 3D) and used ROC analysis to evaluate the diagnostic value of EC strength (Figure 3E). The area under the ROC for aMPFC-ITG connectivity (0.83; 95% confidence interval, 0.70–0.96), PCC-ITG connectivity (0.80; 95% confidence interval, 0.67–0.93), PCC-MFG connectivity (0.74; 95% confidence interval, 0.60–0.88), and PCC-CPL connectivity (0.79; 95% confidence interval, 0.65–0.93) suggested that these connections have clinical diagnostic value for distinguishing SZ patients with AVHs from non-AVHs.

### Validation Results

**FIGURE 4:**
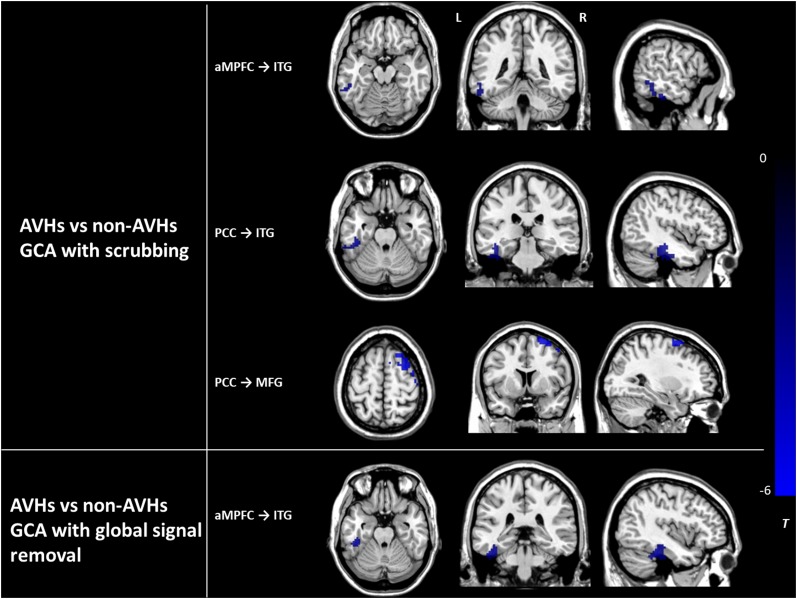
The differences in effective connectivity between the AVHs group and the non-AVHs group with scrubbing and global signal removal, respectively. Color bar represents the *t*-value. PCC, posterior cingulate cortex. aMPFC, anteromedial prefrontal cortex. ITG, inferior temporal gyrus. MFG, middle frontal gyrus. AVHs, auditory verbal hallucinations. L, left. R, right.

The assessment of the effects of GM atrophy and head motion correction and global signal removal on our main findings between the AVHs group and the non-AVHs group showed the following results. (1) The effects of GM loss: There was no significant difference in GMV between the AVHs group and the non-AVHs group, either in local regions or in the whole brain (AVHs: 0.33 ± 0.06 (mean ± SD); non-AVHs: 0.34 ± 0.04; *p* = 0.37). (2) The effects of head motion: Using the statistical analysis accounting for the ‘scrubbing’ procedure in preprocessed images (Power et al., 2012; Yan et al., 2013), we found that the main results in ECs from aMPFC to ITG and from PCC to both ITG and MFG survived (Alphasim corrected *P* < 0.05) (Figure 4). (3) The effects of global signal removal: With the global signal removed, the AVHs group showed significantly decreased EC from aMPFC to ITG (Alphasim corrected *P* < 0.05), which was consistent with our results without removing the global signal. However, the EC from PCC to ITG, MFG, and CPL exhibited non-significant results after global signal removal (Figure 4). It was not solved why the removal of the global signal produced such an effect on EC in this study. This should be further investigated in the future.

## Discussion

In this study, GCA results revealed hypoconnectivity from aMPFC to left ITG and from PCC to left CPL, left ITG, and right MFG in the AVHs group but not in the non-AVHs group. In comparison with HCs, SZ patients with AVHs showed decreased EC from aMPFC to left cingulate gyrus and left ITG and from PCC to bilateral thalamus and increased EC from right MFG, right putamen, and left CPL to aMPFC. The non-AVHs group displayed reduced EC from aMPFC to right cingulate gyrus and left insula and from left superior temporal gyrus to PCC and enhanced EC from left cingulate gyrus, right thalamus, and right putamen to aMPFC relative to HCs. In addition, EC from aMPFC to left ITG was negatively correlated with PANSS positive scores in the two patient groups, and EC from PCC to left ITG was negatively correlated with PANSS negative scores in the non-AVHs group. These findings support our hypothesis and suggest that the information flows of the DMN are relatively disruptive or weaker in SZ patients with AVHs than in those without AVHs, and these changes are correlated with the clinical symptoms of SZ patients. This insight may be helpful to understand the potential neuromechanism underlying AVHs of SZ patients.

Although most rs-fMRI studies have found changes in functional connectivity between DMN and non-DMN brain regions, and also between DMN and other brain networks in SZ patients, the results were somewhat controversial. For example, several studies showed decreased functional connectivity within the DMN in SZ patients compared with HCs (Rotarska-Jagiela et al., 2010; Camchong et al., 2011), while some other studies found increased connectivity within the DMN and between DMN and non-DMN brain regions (Mingoia et al., 2012; Alonso-Solís et al., 2015). Therefore, further studies of functional connectivity are needed to elucidate the role of the DMN in SZ patients. Recently, a spectral DCM study identified reduced EC from the PCC to the anterior frontal node of the DMN in first-episode SZ patients, reflecting the reduced postsynaptic efficacy of prefrontal afferents (Bastos-Leite et al., 2014). Another DCM study in SZ patients found altered EC related to the medial prefrontal cortex, anterior part of the DMN, indicating hippocampal-dorsolateral prefrontal-medial prefrontal hypoconnectivity (Cui et al., 2015). Additionally, SZ patients have shown decreased PCC connectivity with other parts of the DMN compared with HCs, which could then lead to a widespread impairment of communication within the DMN (Bluhm et al., 2007; Rotarska-Jagiela et al., 2010). This study found that, compared with HCs, SZ patients showed significantly altered information flows between the DMN and several SZ-related regions. Specifically, the patient group exhibited decreased efferent information from aMPFC to bilateral cingulate gyrus and from PCC to right MFG and increased afferent information from left cingulate gyrus and right MFG to aMPFC, which were consistent with recent reports on EC of SZ patients (Bastos-Leite et al., 2014; Cui et al., 2015). Additionally, we also found that SZ patients showed abnormal EC between DMN and inferior/superior temporal gyrus, CPL as well as subcortical regions, such as insula, putamen and thalamus, which were not found in the previous studies, but the regions have been consistently reported to be functionally or structurally aberrant in SZ patients in the previous studies (Kuroki et al., 2006; Fornito et al., 2012; Alderson-Day et al., 2015, 2016). Thus, from the perspective of information transmission, these results suggest that the etiology of SZ patients may disrupt the normal processes of DMN network integration and segregation (Karbasforoushan and Woodward, 2012; Alonso-Solís et al., 2015).

With regard to AVH-specific connectivity, our results found that SZ patients with AVHs showed reduced EC from aMPFC to left ITG and from PCC to right MFG, left ITG, and left CPL relative to the non-AVHs group. Similarly, Lawrie et al. (2002) found that the predisposition to hallucinate may be associated with impaired functional connectivity between frontal and temporal brain areas. de la Iglesia-Vaya et al. (2014) uncovered an anomalous process of neural connectivity in the cortico-cerebellar-thalamic-cortical circuits in patients with auditory hallucinations. Additionally, it has been reported that the DMN may be relevant for generating, monitoring, and updating self-referential images, which is possibly based on effective and autobiographical knowledge (Van De Ven, 2013). Early neuropsychological theories have suggested that AVHs might result from a failure in correctly monitoring internally generated speech events (Frith, 2005). A recent review of auditory hallucinations demonstrated that hallucinations were associated with the weaker integrity of the DMN, suggesting that unstable DMN states may be an important precursor to auditory hallucination states (Alderson-Day et al., 2016). Moreover, the interaction of the DMN with other RSNs (Alonso-Solís et al., 2015) and dynamic stability of the DMN are both implicated in hallucinatory states and traits (Jardri et al., 2013). Thus, the aberrant functional architecture of the DMN may contribute to misinterpretations of agency in SZ, which internally generated auditory images or speech and could, in turn, lead to AVHs (Frith, 2005; Wible et al., 2009). Therefore, the findings in this study suggest that the abnormal information flows of the DMN could cause an incorrect monitoring function and interpretation of agency processing in SZ patients with AVHs, which could consequently result in the generation of AVHs in patients.

More importantly, we found that the mean EC from aMPFC to left ITG was negatively correlated with the positive PANSS score, and the mean EC from PCC to left ITG was negatively correlated with the negative PANSS score. Therefore, the left ITG intuitively seems to play a crucial role in the pathogenetic mechanism of AVHs for SZ patients. The ITG locates below the middle temporal gyrus and is connected at the back with the inferior occipital gyrus, which is a part of sensorimotor networks involving auditory and language regions (Alderson-Day et al., 2016). In early studies, the ITG was demonstrated to be probably involved in visual word grapheme system and semantic processing (Nobre et al., 1994; Binder et al., 2000), and it also plays an important role in both dorsal and ventral visual pathways (Sewards and Sewards, 2002). Subsequent studies revealed smaller GMV of bilateral posterior ITG in first-episode and chronic SZ (Onitsuka et al., 2004; Kuroki et al., 2006). Moreover, the GMV of the left posterior ITG was negatively correlated with the factor score for anxiety/depression, and the smaller GMV in the ITG may be related to the pathology common to SZ and affective psychoses (Kuroki et al., 2006). Available evidence suggest that the primary deficit in SZ may be a failure to attenuate precision at the lowest (sensory) cortical levels, leading to a failure of sensory attenuation and characteristic soft neurological signs (e.g., abnormal pursuit eye movements) (Adams et al., 2013). This primary deficit is assumed to induce compensatory increases in the precision of higher levels and consequent difficulties inferring the causes of sensations-causing, for example, hallucinations and delusions (Brown et al., 2013). Therefore, we speculate that the decreased EC from DMN to left ITG and the relation with clinical assessments in this study provide supportive evidence for the assumption that the changes of information from DMN to left ITG could have an effect on the communication between low- and high-level cortical regions in SZ patients and have resulted in the production of AVHs.

Several limitations in this study should be noted. First, the sample size is limited owing to the strict recruitment criteria, including the requirements of drug-naïve and first-episode patients. This could be the reason why the correlation significance failed to survive the FDR correction. A larger sample size is needed for improved statistical power and to further confirm our findings. Second, the seed-based approach was required to manually predefine the seed regions. It could be improved using other analytic methods that overcome this flaw, such as independent component analysis (ICA). However, ICA requires the challenging step of defining the number of components in advance, and using the inappropriate component number in ICA is likely to produce region of interests (ROIs) totally different from what we would like to examine. While every other method bears certain intrinsic shortcomings, we deemed that the seed-based method suited our needs best and, therefore, it was adopted. Finally, the EC analysis in this study only revealed directional functional connectivity. In the future employing a multimodal analysis that exhibits other data modalities, including diffusion tensor imaging, magnetic resonance spectroscopy, electroencephalography, and positron emission tomography, may significantly strengthen the reliability of the findings in this study.

In conclusion, this study has uncovered decreased EC from aMPFC to left ITG and from PCC to left CPL, left ITG, and right MFG in SZ patients with AVHs than those without AVHs using the seed-based GCA approach based on rs-fMRI data. The ROC analysis also shows that the EC in the four brain regions is able to distinguish AVHs patients from non-AVHs patients. More importantly, the mean EC of the left ITG shows significant correlations with clinical assessments in SZ patients. These findings demonstrate that the disrupted information flows of the DMN may cause a failure to internal control on language processing and in turn induced AVHs in SZ patients, and the left ITG plays a vital role in the pathophysiologic mechanism underlying AVHs.

## Author Contributions

DX, MH, and ZZ designed the study. ZZ and DX wrote the manuscript. MH and YX collected imaging and clinical data. XL, GF, ZS, SL, and YX supported the analysis, interpretation, and manuscript revision. All authors contributed to and have approved the final manuscript.

## Conflict of Interest Statement

The authors declare that the research was conducted in the absence of any commercial or financial relationships that could be construed as a potential conflict of interest.

## Footnotes

1 http://www.restfmri.net

2 http://www.fil.ion.ucl.ac.uk/spm
